# Author Response to the Comment on “Time Trends in Cardiovascular Event Incidence in New‐Onset Type 2 Diabetes: A Population‐Based Cohort Study From Germany”

**DOI:** 10.1111/1753-0407.70128

**Published:** 2025-07-23

**Authors:** Theresia Sarabhai, Karel Kostev

**Affiliations:** ^1^ Department of Endocrinology and Diabetology Medical Faculty and University Hospital Düsseldorf, Heinrich‐Heine‐University Düsseldorf Germany; ^2^ Epidemiology IQVIA Frankfurt am Main Germany; ^3^ University Clinic, Philipps, University Marburg Germany

**Keywords:** cardiovascular complications, case‐control study, diabetes mellitus, letter to the editor

Dear Editor,

We sincerely thank the authors of the comment and appreciate the opportunity to respond to clarify specific aspects of our study.

First of all, we agree that laboratory parameters are key indicators of metabolic control and cardiovascular risk. Although the Disease Analyzer database includes laboratory data from a subset of practices, laboratory values were not consistently available over time and across patients in our cohort. Therefore, we opted not to include them in our analyses. To address this limitation, we adjusted for chronic comorbidities known to be associated with poor metabolic control, such as hypertension, dyslipidemia, and obesity (coded diagnoses). Previous studies demonstrated the validity of the Disease Analyzer database especially for case–control studies focusing on diabetes mellitus [1]. Furthermore, by focusing on first cardiovascular events in a well‐defined incident T2D cohort without prior CVD, we aimed to reduce confounding and improve internal validity despite the absence of uniformly available laboratory markers.

We acknowledge the potential limitations of relying solely on ICD‐10 codes, as we stated in the manuscript. However, the Disease Analyzer database has been extensively validated and has demonstrated a high positive predictive value for major cardiovascular diagnoses, including MI [1, 2, 3]. We appreciate the reference by Tsai et al. [4], which highlights the strong validity of ICD‐10‐CM codes for identifying AMI subtypes. Although their work confirms excellent performance metrics, our interest was not the subtype but rather the trend in overall MI incidence.

Indeed, smoking is a critical risk factor. As stated, individual smoking status is not recorded in the Disease Analyzer database. We therefore followed established practice in using COPD as a proxy, recognizing its limitations. As noted in the literature, around 90% of patients with COPD are current or former smokers [5]. However, not all smokers develop COPD, so COPD cannot fully replace smoking status, but provides an approximate indicator. We clearly acknowledged this limitation in the manuscript. Importantly, smoking prevalence in Germany has declined over the study period, which may have contributed to overall cardiovascular improvements. However, as these trends would influence both diabetic and nondiabetic populations alike, our interpretation focused on diabetes‐specific outcomes in a matched T2D cohort.

We agree that socioeconomic status and medication use are important factors. Although these data were not available in sufficient detail in our database, our large, matched cohorts and adjustment for key comorbidities offer valuable insights into temporal patterns. The unchanged MI and IS incidence, despite improvements in CHD and TIA, likely reflects a balance between earlier vascular damage and therapeutic progress. Although we did not stratify events by time since diagnosis, our 5‐year follow‐up captures overall incidence patterns. The rise in hypertension and obesity may have offset some benefits of improved care. We do not infer causality but interpret our findings as temporal trends, acknowledging that CHD and TIA declines are likely multifactorial—driven by both clinical improvements and population‐level changes.

Despite limitations, our study provides novel insights into cardiovascular trends in incident T2D. We welcome further research incorporating lifestyle, biomarker, and socioeconomic status data to extend these findings.

With kind regards,

Theresia Sarabhai and Karel Kostev.

## Author Contributions

In accordance with the ICMJE guidelines, the individual contributions are as follows: T.S. and K.K. manuscript drafting, and response coordination. All authors have read and approved the final version of the response and agree to be accountable for all aspects of the work.

## Disclosure

The authors have nothing to report.

## Conflicts of Interest

The authors declare no conflicts of interest.
